# A pooled prevalence of fetal alcohol spectrum disorders in South Africa: a systematic review and meta-analysis protocol

**DOI:** 10.1186/s13690-021-00679-0

**Published:** 2021-08-30

**Authors:** Babatope O. Adebiyi, Ferdinand C. Mukumbang

**Affiliations:** 1 Centre for Interdisciplinary Studies of Children, Families and Society, University of the Western Cape, Robert Sobukwe Road, Bellville, Cape Town, 7535 South Africa; 2 Department of Global Health, Schools of Medicine and Public Health, University of Washington, Seattle, WA USA

**Keywords:** Fetal alcohol Spectrum disorder, Prevalence, Systematic review, South Africa, Meta-analysis, Neurobehavioral disorder associated with prenatal alcohol exposure

## Abstract

**Background:**

Fetal Alcohol Spectrum Disorder (FASD) remains a global public health problem. South Africa is estimated to have the highest recorded prevalence of FASD. However, no study has systematically evaluated the available prevalence studies to provide estimates that may facilitate effective planning and delivery of prevention and management services. Therefore, we propose to conduct a systematic review and meta-analysis to report a pooled estimate of the FASD prevalence among children, youth and adults in South Africa.

**Methods:**

We will include quantitative (cohort and cross-sectional) studies that reported on the prevalence of FASD in South Africa. We will search databases such as Academic Search Complete, Education Resource Information Center (ERIC), SocINDEX, Health Source: Nursing/Academic Edition, Cumulative Index of Nursing and Allied Health and PsycARTICLES), Scopus, Science Direct, Springer Link, JSTOR, SAGE journals, PubMed, Web of Science and Sabinet. The references of included studies will be searched for additional studies on the prevalence of FASD. The search will be from inception to October 2021. Screening of (titles, abstracts and full text of the potentially relevant articles) will be done by two independent authors using software. All disagreements will be resolved by discussion. A standardised data extraction form will be designed for the extraction. Two authors will independently extract the data from the selected articles and all disagreements will be resolved by discussion. We will use a tool developed by Munn and colleagues to critically appraise all the included studies. The primary outcome will be the proportion of individuals with FASD in South Africa. We will use the Freeman–Tukey double arcsine transformation to transform the raw prevalence estimates so that the data can follow an approximately normal distribution. We will use random-effects models to calculate 95% confidence intervals and prediction intervals based on multiple meta-analyses with transformed proportions. We will test heterogeneity using Cochran’s Q and describe using the I^2^ statistic.

**Discussion:**

The pooled prevalence estimate will assist the government and other stakeholders (such as non-profit organisations and researchers) to plan and prioritise prevention and management interventions.

**Systematic review registration:**

The protocol has been registered with PROSPERO (registration number: CRD42020197979).

## Background

Alcohol is identified as the primary cause of preventable birth defects and developmental disorders [1]. Alcohol consumption during pregnancy may lead to an array of disorders collectively known as Fetal Alcohol Spectrum Disorder (FASD) [2–4]. To be diagnosed with any of the FASD (Fetal Alcohol Syndrome {FAS}, Partial FAS, {PFAS}, Alcohol-Related Neurodevelopmental Disorder {ARND}, and Alcohol-Related Birth Defects {ARBD}), an individual must meet all or some of the following criteria [2–4]. These criteria comprise documented prenatal alcohol exposure, pre-and post-natal growth deficiency, deficient brain growth and neurobehavioral impairment – with or without cognitive impairment [2–4]. In addition to the above-mentioned diagnostic terms, “Neurobehavioral Disorder Associated with Prenatal Alcohol Exposure” (ND-PAE) has been proposed and included in the 2013 Diagnostic and Statistical Manual for Mental Health Disorders (DSM-5) [5–8].

According to the World Health Organization, South Africa has one of the highest reported per capita rates of alcohol consumption in the world among those who do consume alcohol [9]. It is widely held that heavy drinking among poorer South Africans is deeply rooted in the legacy of the ‘dop’ system, whereby alcoholic beverages were offered to farmworkers as part of their wages [10]. Alcohol consumption during pregnancy is also widespread in South Africa at a rate ranging from 2.5 to 45% as reported in a review of alcohol exposure among pregnant women in sub-Saharan Africa [11]. An estimated 36.9% of women consume alcohol during pregnancy or in the last 3 months before they become aware of their pregnancy in a study conducted in the Western Cape Province [12].

One in every 13 alcohol-exposed pregnancies results in a FASD [13]. Globally, about 630,000 children are born with FASD each year [13]. The global prevalence of FASD was estimated to be 8 per 1000 children and youth in 2017 [13]. The prevalence of FASD varies from one country to another and in most countries, it has only been estimated among sub-groups of the general population. For example, in Canada, the prevalence of FASD was estimated to be 18.1 per 1000 children in 2019 [14]. In 2018, the prevalence of FASD was reported to range from 11.3 to 50.0 per 1000 children in the United States of America (USA) [15]. In Australia, the prevalence of FASD was to be 194.4 per 1000 children in 2017 [16].

According to the Foundation for Alcohol Related Research (FARR), about 6 million individuals are affected by FASD in South Africa [17]. In a prevalence study conducted in some selected provinces of South Africa, the prevalence of FASD was estimated to range from 29 to 290 per 1000 live births in 2016 [18]. The prevalence of FASD varies from one province to another within South Africa. In 2017, the prevalence of FASD among grade one pupils in the Western Cape was estimated to be 196 to 276 per 1000, the highest recorded prevalence in any part of the world [19]. In the Northern Cape, the prevalence was estimated at 63.9 per 1000 grade one pupils in 2015 [20].

Prevalence studies have been conducted in South Africa, however, most of the studies have been conducted among subgroups of the general population and in certain provinces. The evidence obtained from these prevalence studies at best informs provincial and regional policies and interventions. A pooled estimate of FASD prevalence will provide robust evidence to plan FASD-related prevention, treatment and management services at the national level through the design and implementation of national policy, which are currently non-existing [21].

This proposed systematic review with meta-analysis will paint a better picture of the problem of FASD in South Africa. In addition, the result of the review will provide information that may be used to lobby policymakers for the prioritisation of the FASD issues (which is currently limited) in policies and interventions. Moreover, the pooled estimate will facilitate an accurate comparison of the prevalence of FASD in South Africa to other countries. Therefore, we propose to conduct a systematic review and meta-analysis to report a pooled estimate of the FASD prevalence in South Africa.

### Research question

What is the pooled prevalence of FASD among children, youth and adults in the general population in South Africa?

## Methods

In reporting this study, we will follow the Preferred Reporting Items for Systematic Reviews and Meta-Analyses Protocols (PRISMA-P) checklist [22] and Meta-analysis of Observational Studies in Epidemiology (MOOSE) reporting guidelines [23]**.** This protocol has been registered in the International Prospective Register of Systematic Reviews (PROSPERO) database (registration number: CRD42020197979).

### Search strategy

We will search databases such as Academic Search Complete, Education Resource Information Center (ERIC), SocINDEX, Health Source: Nursing/Academic Edition, Cumulative Index of Nursing and Allied Health, PsycARTICLES, Scopus, Science Direct, Springer Link, JSTOR, SAGE journals, PubMed and Sabinet. The grey literature will also be searched. The references of included studies will be searched for additional studies on the prevalence of FASD. In addition, experts in the field will be contacted for recent studies. The search will be from inception (January 1973, when FAS was first described) to October 2021.

### Search terms

The search terms will include Fetal Alcohol Spectrum Disorders or Fetal Alcohol Syndrome or Partial Fetal Alcohol Syndrome or Alcohol-Related Birth Defects or Alcohol-Related Neurodevelopment Disorder or Fetal Alcohol Effects (FAE) or Prenatal Alcohol Exposure (PAE) or Neurobehavioral Disorder Associated with Prenatal Alcohol Exposure (ND-PAE) and epidemiology or frequency or incidence or prevalence and South Africa. We will combine the search terms in various forms for the selected databases using Boolean operators. The search will be done by one of the authors with the assistance of a University librarian and the second author will check the results of the search for accuracy. Both authors have expertise in conducting systematic reviews and meta-analyses. The combination of search terms for one of the databases is presented in Table 1 below.

**Table 1:** Combination of terms

Keywords combinations	
(1) Prevalence	- F*tal alcohol spectrum disorder or FASD AND Prevalence AND South Africa - F*tal alcohol syndrome or FASD AND Prevalence AND South Africa - Partial f*tal alcohol syndrome or PFAS AND Prevalence AND South Africa - Prenatal alcohol exposure or pre-natal alcohol exposure or PAE AND Prevalence AND South Africa - Neurobehavioral disorder associated with prenatal alcohol exposure or ND-PAE AND Prevalence AND South Africa - Alcohol related neurodevelopmental disorder or ARND AND Prevalence AND South Africa - F*tal alcohol effect or FAE AND Prevalence AND South Africa
(2) Epidemiology	- F*tal alcohol spectrum disorder or FASD AND Epidemiology AND South Africa - F*tal alcohol syndrome or FASD AND Epidemiology AND South Africa - Partial f*tal alcohol syndrome or PFAS AND Epidemiology AND South Africa - Prenatal alcohol exposure or pre-natal alcohol exposure or PAE AND Epidemiology AND South Africa - Neurobehavioral disorder associated with prenatal alcohol exposure or ND-PAE AND Epidemiology AND South Africa - Alcohol related neurodevelopmental disorder or ARND AND Epidemiology AND South Africa - F*tal alcohol effect or FAE AND Epidemiology AND South Africa
(3) Frequency	- F*tal alcohol spectrum disorder or FASD AND Frequency AND South Africa - F*tal alcohol syndrome or FASD AND Frequency AND South Africa - Partial f*tal alcohol syndrome or PFAS AND Frequency AND South Africa - Prenatal alcohol exposure or pre-natal alcohol exposure or PAE AND Frequency AND South Africa - Neurobehavioral disorder associated with prenatal alcohol exposure or ND-PAE AND Frequency AND South Africa - Alcohol related neurodevelopmental disorder or ARND AND Frequency AND South Africa - F*tal alcohol effect or FAE AND Frequency AND South Africa
(4) Incidence	- F*tal alcohol spectrum disorder or FASD AND Incidence AND South Africa - F*tal alcohol syndrome or FASD AND Incidence AND South Africa - Partial f*tal alcohol syndrome or PFAS AND Incidence AND South Africa - Prenatal alcohol exposure or pre-natal alcohol exposure or PAE AND Incidence AND South Africa - Neurobehavioral disorder associated with prenatal alcohol exposure or ND-PAE AND Incidence AND South Africa - Alcohol related neurodevelopmental disorder or ARND AND Incidence AND South Africa - F*tal alcohol effect or FAE AND Incidence AND South Africa

### Eligibility criteria

Studies will be considered for eligibility based on the following criteria.

#### Type of population/participants

We will include studies that recruited children, youth and adults. In addition to the above, studies must include population and participants from South Africa. We will not place any restrictions on the age of participants. Studies with individuals without FASD and those from other countries will be excluded. Also, all animal studies will be excluded.

### Types of outcomes

The outcome is the proportion of individuals with FASD (FAS, PFAS, ARND and ARBD) per thousand. Studies will be included whether or not an outcome of interest is reported as a primary or secondary outcome in the original article.

### Type of studies/study designs

We will include quantitative (cohort {prospective and retrospective} and cross-sectional) studies that reported on the prevalence of FASD (FAS, PFAS, ARND and ARBD) in South Africa. We will include studies if they (1) comprised of original research that estimated the prevalence of FASD; (2) presented the results of primary data; (3) are published in peer-reviewed journals; (4) written in the English language; (5) provided the prevalence of FASD with a measure of uncertainty or the necessary information to calculate uncertainty (such as sample size or the number of cases); (6) specified the diagnostic guideline or case definition used for the estimate of the prevalence and (7) used active case ascertainment (searching for individuals with FASD in selected populations), clinic-based methods (estimation of FASD prevalence based on populations seen in hospitals or clinics) and passive surveillance (estimation of FASD prevalence via existing records and registries). We will exclude studies if they (1) consisted of reviews (scoping, systematic, narrative and rapid) and (2) reported a pooled estimate by combining several studies.

### Screening of studies

The screening will be done based on our eligibility criteria set at the beginning of the current study. The screening processes will be conducted using software called “Rayyan” - a free webtool designed to help researchers working on systematic reviews [24]. The results of all the searches will be imported into the software and duplicates will be removed. After that, two authors will carry out the screening independently (title, abstract and full-text) using the software and the disagreements will be resolved through discussion.

### Data extraction

A standardised data extraction form will be designed for the extraction of the information of interest from each study. Two authors will independently extract the data from the selected articles and all disagreements will be resolved by discussion. We will extract the following variables from the identified studies: province, study year(s), sample size, number of cases, prevalence, 95% CI, age range, percentage of male or female participants in the sample, method of ascertainment, the diagnostic guideline or case definition used and the proportion of potentially eligible people.

### Critical appraisal of identified studies

We will critically appraise all the included studies using a tool developed by Munn et al. [25] for the methodological guidance for systematic reviews of observational epidemiological studies reporting prevalence and incidence data. The tool consists of nine criteria:

1. Was the sample frame appropriate to address the target population?
2. Were study participants sampled appropriately?
3. Was the sample size adequate?
4. Were the study subjects and the setting described in detail?
5. Was the data analysis conducted with sufficient coverage of the identified sample?
6. Were valid methods used for the identification of the condition?
7. Was the condition measured in a standard, reliable way for all participants?
8. Was there appropriate statistical analysis?
9. Was the response rate adequate, and if not, was the low response rate managed appropriately?

In addition, the certainty of the evidence will be assessed using the Grading of Recommendations, Assessment, Development and Evaluations (GRADE) tool [26]. The following domains of GRADE will be assessed: risk of bias, imprecision, inconsistency, indirectness and publication bias. The overall strength of evidence of the outcome of interest will be reported. The levels of quality of evidence will be placed into one of the four categories: very low, low, moderate and high. Two authors will independently appraise all the included studies and disagreements will be resolved by discussion.

### Meta-analyses

To estimate the pooled prevalence of FASD in South Africa, we will conduct a meta-analysis assuming the presence of heterogeneity a priori and use a random-effects model to analyse the results. We will transform the data using the Freeman-Tukey double arcsine transformation to prevent the overweighting of studies reporting extremely low prevalence. We will assess heterogeneity between prevalence estimates using the Cochrane Q test and the *I*^2^ statistic. We will assess publication bias by (1) visually inspecting the funnel plot for a skewed distribution; (2) by using a ranked correlation test and (3) a weighted regression test. We will access publication bias (if it is present because it is unlikely in the case of prevalence studies) by the inspection of Funnel plots.

Individual and pooled estimates from all meta-analyses will be presented using tables, and forest plots will be created. Statistical heterogeneity will be measured and evaluated using forest plots and calculations of Cochran’s chi-square test and the I^2^ statistic during meta-analyses, and guidance from the Cochrane Handbook will be used to interpret heterogeneity.

A narrative synthesis will be used if meta-analysis is not suitable.

## Discussions

In South Africa, several studies have reported a lack of specific policies for the prevention and management of FASD [27–31]. However, clauses that could be attributed to the prevention and management of FASD exist in other related policy documents [21]. Also, a review has reported the scant prevention and management interventions for FASD in South Africa [32]. The lack of a specific policy and the scanty existence of interventions for FASD could be because there are several public health issues demanding policy attention and interventions. Because of limited resources, the government will need to set priorities for public health policy, funding for public health initiatives and healthcare planning. Therefore, the government needs research-based evidence on each issue to plan intervention strategies.

### Ethics and dissemination

This study (protocol) does not require ethical clearance. It does not involve participants, therefore, there are no rights or privacy to protect. According to the PRISMA statement and checklist, the study will involve searching for the published papers and analysing their outcomes. After the completion of both the protocol and the review, they will be published in peer-reviewed journals to disseminate the findings widely. Also, the findings will be shared in non-peer review platforms such as the conversation and online newspapers (News24, Daily Maverick and Mail and Guardian).

## Data Availability

Not Applicable.

## Abbreviations

FASD: Fetal Alcohol Spectrum Disorder
FAS: Fetal Alcohol Syndrome
PFAS: Partial FAS
ARBD: Alcohol-Related Neurodevelopmental Disorder (ARND), and Alcohol-Related Birth Defects
ND-PAE: Neurobehavioral Disorder Associated with Prenatal Alcohol Exposure
DSM-5: Diagnostic and Statistical Manual for Mental Health Disorders
PRISMA-P: The Preferred Reporting Items for Systematic Reviews and Meta-Analyses Protocols
MOOSE: Meta-analysis of Observational Studies in Epidemiology
PROSPERO: International Prospective Register of Systematic Reviews
USA: United State of America
FARR: Foundation for Alcohol Related Research
WHO: World Health Organization
GRADE: Grading of Recommendations, Assessment, Development and Evaluations

## References

[1] Bailey BA, Sokolo RJ (2008). Pregnancy and alcohol use: evidence and recommendations for prenatal care. Clin Obstet Gynecol.

[2] Cook JL, Green CR, Lilley CM (2016). Fetal alcohol spectrum disorder: a guideline for diagnosis across the lifespan. CMAJ.

[3] Chudley AE, Conry J, Cook JL (2005). Fetal alcohol Spectrum disorder: Canadian guidelines for diagnosis. CMAJ.

[4] Hoyme HE, Kalberg WO, Elliott AJ (2016). Updated clinical guidelines for diagnosing fetal alcohol spectrum disorders. Pediatrics.

[5] Kable JA, Mukherjee RAS (2017). Neurodevelopmental disorder associated with prenatal exposure to alcohol (ND-PAE): a proposed diagnostic method of capturing the neurocognitive phenotype of FASD. Eur J Med Genet.

[6] Kable JA, O’Connor MJ, Olson HC (2016). Neurobehavioral disorder associated with prenatal alcohol exposure (ND-PAE): proposed DSM-5 diagnosis. Child Psychiatry Hum Dev.

[7] Brown JM, Bland R, Jonsson E (2019). The standardization of diagnostic criteria for fetal alcohol Spectrum disorder (FASD): implications for research, clinical practice and population health. Can J Psychiatr.

[8] Olson HC (2015). Advancing recognition of fetal alcohol Spectrum disorders: the proposed DSM-5 diagnosis of “neurobehavioral disorder associated with prenatal alcohol exposure (ND-PAE)”. Curr Dev Disord Reports.

[9] World Health Organisation. Global status report on alcohol and health. World Heal Organ; 122, http://www.who.int/substance_abuse/publications/global_alcohol_report/en/ (2011, Accessed 27 June 2020).

[10] May PA, Marais AS, De Vries M, et al. The dop system of alcohol distribution is dead, but it’s legacy lives on. Int J Environ Res Public Health. 2019;16. 10.3390/ijerph16193701 Epub ahead of print. 10.3390/ijerph16193701 PMC6801681 31581441

[11] Culley CL, Ramsey TD, Mugyenyi G (2013). Alcohol exposure among pregnant women in sub-saharan Africa: a systematic review. J Popul Ther Clin Pharmacol.

[12] Petersen Williams P, Jordaan E, Mathews C (2014). Alcohol and other drug use during pegnancy among women attending midwife obstetric units in the cape Metropole, South Africa. Adv Prev Med.

[13] Lange S, Probst C, Gmel G (2017). Global prevalence of fetal alcohol Spectrum disorder among children and youth. JAMA Pediatr.

[14] Popova S, Lange S, Poznyak V (2019). Population-based prevalence of fetal alcohol spectrum disorder in Canada. BMC Public Health.

[15] May PA, Chambers CD, Kalberg WO (2018). Prevalence of fetal alcohol spectrum disorders in 4 US communities. JAMA.

[16] Fitzpatrick JP, Latimer J, Olson HC (2017). Prevalence and profile of neurodevelopment and fetal alcohol spectrum disorder (FASD) amongst Australian Aboriginal children living in remote communities. Res Dev Disabil.

[17] Foundation for Alcohol Related Research (FARR). What we Do - FARR SA, https://www.farrsa.org.za/what-we-do/ (2018, Accessed 16 July 2018).

[18] Olivier L, Curfs LMG, Viljoen DL (2016). Fetal alcohol spectrum disorders: prevalence rates in South Africa. South African Med J.

[19] May P, De Vries M, Marais A-S (2017). Replication of high fetal alcohol spectrum disorders prevalence rates, child characteristics, and maternal risk factors in a second sample of rural communities in South Africa. Int J Environ Res Public Health.

[20] Urban MF, Olivier L, Viljoen D (2015). Prevalence of fetal alcohol syndrome in a south African city with a predominantly black African population. Alcohol Clin Exp Res.

[21] Adebiyi BO, Mukumbang FC, Beytell A-M (2019). To what extent is fetal alcohol Spectrum disorder considered in policy-related documents in South Africa? A document review. Heal Res Policy Syst.

[22] Moher D, Shamseer L, Clarke M (2016). Preferred reporting items for systematic review and meta-analysis protocols (PRISMA-P) 2015 statement. Rev Esp Nutr Humana y Diet.

[23] Stroup DF, Berlin JA, Morton SC (2000). Meta-analysis of observational studies in epidemiology: a proposal for reporting. J Am Med Assoc.

[24] Morris M. Guides: Rayyan for Systematic Reviews: Getting started, https://libraryguides.mcgill.ca/rayyan/home (2020, Accessed 18 Mar 2021).

[25] Munn Z, Moola S, Lisy K (2015). Methodological guidance for systematic reviews of observational epidemiological studies reporting prevalence and cumulative incidence data. Int J Evid Based Healthc.

[26] Iorio A, Spencer FA, Falavigna M (2015). Use of GRADE for assessment of evidence about prognosis : rating confidence in estimates of event rates in broad categories of patients.

[27] Adebiyi BO, Mukumbang FC, Cloete LG, et al. Exploring service providers’ perspectives on the prevention and management of fetal alcohol spectrum disorders in South Africa: A qualitative study. BMC Public Health. 2018. Epub ahead of print;18. 10.1186/s12889-018-6126-x. 10.1186/s12889-018-6126-x PMC6220472 30400900

[28] Adebiyi BO, Mukumbang FC, Okop KJ (2018). A modified Delphi study towards developing a guideline to inform policy on fetal alcohol spectrum disorders in South Africa: a study protocol. BMJ Open.

[29] Adebiyi BO, Mukumbang FC, Cloete LG, et al. Policymakers’ perspectives towards developing a guideline to inform policy on fetal alcohol spectrum disorder: a qualitative study. Int J Environ Res Public Health. 2019. Epub ahead of print;16. 10.3390/ijerph16060945. 10.3390/ijerph16060945 PMC6466131 30884766

[30] Adebiyi BO, Mukumbang FC, Beytell AM. A guideline for the prevention and management of fetal alcohol spectrum disorder in South Africa. BMC Health Serv Res. 2019. Epub ahead of print;19. 10.1186/s12913-019-4677-x. 10.1186/s12913-019-4677-x PMC6836420 31694624

[31] Adebiyi BO, Mukumbang FC, Beytell AM. Policy Requirements for the Prevention and Management of Fetal Alcohol SpectrumDisorder in South Africa: A Policy Brief. Frontiers in Public Health. 2021;9:368. 10.3389/fpubh.2021.592726 PMC8082060 33937161

[32] Adebiyi BO, Mukumbang FC, Erasmus C (2019). The distribution of available prevention and management interventions for fetal alcohol Spectrum disorder (2007 to 2017): implications for collaborative actions. Int J Environ Res Public Health.

